# Base excess can be misleading in acute respiratory acidosis

**DOI:** 10.1186/cc9556

**Published:** 2011-03-11

**Authors:** S Kocsi, K Kiss, B Szerdahelyi, M Demeter, Z Molnar

**Affiliations:** 1 University of Szeged, Hungary

## Introduction

Base excess (BE) is the measure of nonrespiratory change of acid-base status in the body. It is calculated after correcting the blood sample's pH to 7.4, temperature to 37°C and pCO_2 _to 40 mmHg. Actual HCO_3 _level is a metabolic parameter derived directly from the Henderson-Hasselbalch equation. There is some evidence that temporary changes in pCO_2 _affect BE [1,2], but little is known about the response of HCO_3_. Therefore, the aim of this study was to investigate the relationship between BE and HCO_3 _in critically ill patients immediately after admission to the ICU.

## Methods

The first arterial blood gas samples (within 1 hour of admission) of patients admitted to our ICU were retrospectively evaluated and pH, HCO_3_, pCO_2 _and BE were registered and analysed. After testing the data distribution, correlation was determined with Pearson's correlation.

## Results

Arterial blood gas samples from 88 patients were analysed. There was a strong, significant correlation between BE and HCO_3 _(*r*^2 ^= 0.93, *P *< 0.001) in the whole sample. In blood samples with pCO_2 _> 45 mmHg, in 26 cases the pH was >7.3, and in 15 cases pH was <7.3 (that is, acute respiratory acidosis). In these cases with a cut-off BE <0 mmol/l, the BE had sensitivity = 73% and specificity = 85% for predicting acidosis. With a cut-off for HCO_3 _< 24 mmol/l, the HCO_3 _had sensitivity = 27% and specificity = 100% for acidosis. Choosing a cut-off for BE <-2 mmol/l, sensitivity = 47%, specificity = 100%; for HCO_3 _< 22 mmol/l, sensitivity = 13%, specificity = 100%.

## Conclusions

Although BE and HCO_3 _had very good correlation in the whole sample, in acute respiratory acidosis BE indicated metabolic acidosis with high sensitivity, while the high specificity and low sensitivity of HCO_3 _showed that there was no metabolic component of the acid-base imbalance. Therefore, in accord with previous studies, our preliminary results give further evidence that HCO_3 _is a more reliable parameter to analyse acid-base balance in acute circumstances, especially in acute respiratory acidosis, than BE.
